# Regulation of Intestinal Epithelial Cells Properties and Functions by Amino Acids

**DOI:** 10.1155/2018/2819154

**Published:** 2018-05-09

**Authors:** Shanshan Kong, Yanhui H. Zhang, Weiqiang Zhang

**Affiliations:** ^1^Department of Pediatrics, College of Medicine, University of Tennessee Health Science Center, Memphis, TN 38163, USA; ^2^Department of Bioscience Research, College of Dentistry, University of Tennessee Health Science Center, Memphis, TN 38163, USA; ^3^Department of Physiology, College of Medicine, University of Tennessee Health Science Center, Memphis, TN 38163, USA

## Abstract

Intestinal epithelial cells (IECs) line the surface of intestinal epithelium, where they play important roles in the digestion of food, absorption of nutrients, and protection of the human body from microbial infections, and others. Dysfunction of IECs can cause diseases. The development, maintenance, and functions of IECs are strongly influenced by external nutrition, such as amino acids. Amino acids play important roles in regulating the properties and functions of IECs. In this article, we briefly reviewed the current understanding of the roles of amino acids in the regulation of IECs' properties and functions in physiological state, including in IECs homeostasis (differentiation, proliferation, and renewal), in intestinal epithelial barrier structure and functions, and in immune responses. We also summarized some important findings on the effects of amino acids supplementation (e.g., glutamine and arginine) in restoring IECs' and intestine functions in some diseased states. These findings will further our understanding of the important roles of amino acids in the homeostasis of IECs and could potentially help identify novel targets and reagents for the therapeutic interventions of diseases associated with dysfunctional IECs.

## 1. Introduction

### 1.1. Digestive System, the Structure, and Functions of Intestine

The human body has several levels of organization: cells, tissues, organs, and organ systems. Working together, human organ systems supply the body's cells with essential biological materials they need to function, as well as facilitating the elimination of wastes. They also work in a coordinated fashion to maintain temperature, pH, and other conditions at optimal levels to support cellular processes. The digestive system has three main functions: digestion of food, absorption of nutrients, and elimination of solid food waste. The organs of digestive system include those that make up the gastrointestinal (GI) tract (e.g., esophagus, stomach, and small and large intestines) and accessory organs (e.g., liver, gall bladder, and pancreas).

The small and large intestines form a major part of the GI tract. Located in the abdomen, they are the major site of digestion and absorption of nutrients from ingested food [1]. The small intestine consists of three sections: duodenum, jejunum, and ileum [2]. The duodenum is the first and the shortest segment of small intestine. It receives partially digested foods from stomach and pancreatic secretions containing digestive enzymes; it plays important roles in digesting foods. The jejunum lies in the midsection of intestine, connecting the duodenum and ileum. It contains circular folds and villi to increase surface area for absorbing small nutrient particles that were enzymatically digested in the duodenum. The absorbed nutrients then enter liver through the enterohepatic circulation. The ileum is the third part of small intestine and contains villi similar to those in jejunum. The ileum absorbs vitamin B12, bile acids, and other remaining nutrients not absorbed by jejunum [2]. Villi are absent in caecum and large intestine (colon).

**Figure 1:**
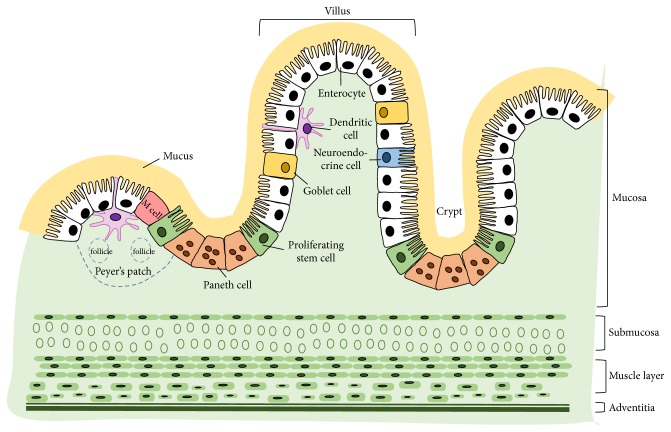
A cartoon depicting the cross-sectional structure of small intestine and the major cell constituents of epithelium. The small intestine consists of four layers: mucosa, submucosa, muscle layer, and adventitia. The intestinal epithelium is lined with a single layer of polarized cells, among which the major types include enterocytes, goblet cells, Paneth cells, stem cells, and others.

The cross-sectional structure of small intestine contains four layers: mucosa, submucosa, muscular layer, and adventitia [3] (Figure 1). The mucosa consists of epithelial cells, which secrete mucus as a thick protective fluid. The main functions of the mucosa are to absorb and transport nutrients, keep the tissues moist, and protect the body from pathogens and foreign particles [4]. The submucosa is a relatively thin, collagen-rich extracellular matrix, which supports the mucosa and joins it to the muscular layer. The muscular layer consists of muscle tissue; it is responsible for gut movement such as peristalsis [3]. The adventitia is a smooth tissue membrane consisting of two layers of mesothelium, a visceral membrane and a parietal layer. The adventitia secretes serous fluid [3].

### 1.2. The Properties and Functions of Intestinal Epithelial Cells (IECs)

IECs exist as a layer of cells that line the luminal surface of intestinal epithelium. IECs are continuously replaced every 4-5 days through a process of renewal and migration. New IECs are produced by stem cells located in crypts at the base of the intestinal glands. These stem cells give rise to progenitors that differentiate into mature IECs types as they (except Paneth cells) migrate up the crypt-villus axis [5]. The aged IECs undergo apoptosis and are shed off into intestinal lumen. Several cell types are present in the intestinal epithelium, including enterocytes, Paneth cells, goblet cells, and neuroendocrine cells (Figure 1).

Enterocytes are the major cell type in intestinal epithelium. They are simple columnar epithelial cells and play important roles in nutrient absorption (e.g., ions, water, sugar, peptides, and lipids) and in secreting immunoglobulins. Goblet cells comprise around 10% of all IECs. Goblet cells secrete mucus, which lubricates the passage of food through the intestines and protects the intestinal wall from digestive enzymes [6]. Paneth cells are only found in small intestine, particularly in the ileum [7]. Paneth cells synthesize and secrete antimicrobial peptides and proteins. It was reported that Paneth cells can directly sense enteric bacteria via cell-autonomous MyD88-dependent toll-like receptor (TLR) activation, triggering expression of multiple antimicrobial factors [8]. Paneth cells are long-lived cells; they migrate downwards to the base of crypts after differentiating from stem cells. Neuroendocrine cells can release intestinal hormones or peptides into bloodstream upon stimulation, to activate nervous responses [9]. Neuroendocrine cells are also known to act as chemoreceptors, initiating digestive actions, detecting harmful substances, and initiating protective responses [10].

Other cell types reported include exocrine cells and endocrine cells. Exocrine cells are located in the mucosa of small intestine; they secrete mucus, peptidase, sucrose, maltase, lactase, lipase, and enteropeptidase [9]. Endocrine cells secrete cholecystokinin and secretin. Their secretions are primarily regulated by chyme: the larger the amount of chyme present, the greater the secretions [11].

## 2. Amino Acids Regulate the Properties and Functions of IECs

### 2.1. The Absorption of Amino Acids in the Intestine

According to the National Academies of Sciences Engineering Medicine, the Dietary Reference Intake is 0.8 grams of protein per kilogram of body weight [12]. Most of these proteins cannot be absorbed by human body in their predigested state and must be converted into amino acids prior to absorption [13]. Digestion describes the process by which complex dietary substances are converted into simple forms that can be absorbed by the body. Protein digestion starts in stomach and finishes in the intestine. Three enzymes are important for protein digestion: pepsin, trypsin, and chymotrypsin [14]. The acidic environment in stomach denatures proteins and makes them available for proteolytic digestion. Pepsin is the major proteolytic enzyme in stomach, where it converts large proteins into smaller peptides. The partially digested peptides enter small intestine, where they are further degraded by activated trypsin and chymotrypsin. Trypsin and chymotrypsin are secreted by pancreas as inactive forms and converted into active forms when secreted into small intestine [14]. Proteins digestion can be further enhanced by proteases, such as aminopeptidase N. Aminopeptidases can digest proteins from the amino terminus and form single amino acids or di- and tripeptides [15].

**Figure 2:**
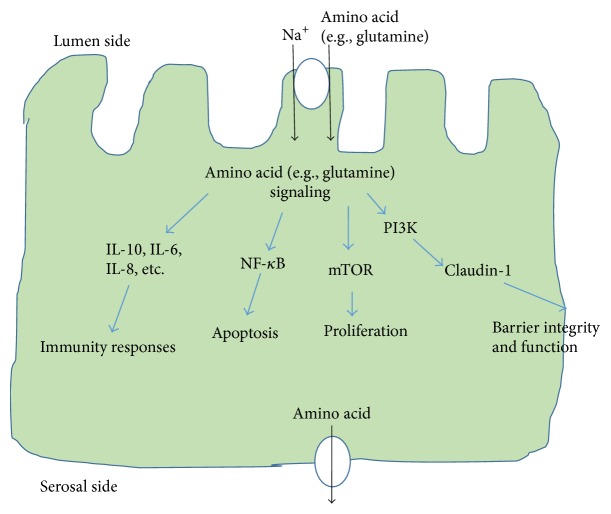
A schematic view of the absorption of amino acids in IECs and some regulatory roles of amino acids in IECs' properties and functions. Amino acids can be transported into IECs by transporters (e.g., sodium coupled neutral amino transporter 2) at the luminal plasma membrane and out of IECs by transporters at the serosal side (and then into blood vessels for circulation). Amino acids regulate a wide range of IECs' properties and functions through diverse signaling pathways. In this article, we focus on discussing the roles of amino acids (especially glutamine and arginine) on the proliferation, barrier function, and immunity responses of IECs. Some of the involved signaling pathways are depicted here and detailed discussions are provided in the text. mTOR: mammalian target of rapamycin; NF-*κ*B: nuclear factor kappa-light-chain-enhancer of activated B cells; PI3K: phosphatidylinositide 3-kinases; IL-6: interleukin 6; IL-8: interleukin 8; IL-10: interleukin 10.

Absorption of amino acids is mainly carried out by IECs through active transport [16]. These IECs are highly polarized, with the apical plasma membrane facing the intestinal lumen. Because there are different types of transporters localized in either apical or basolateral membranes, IECs can transport substances in one direction across the epithelium. There are more than 7 types of amino acids transporters in the apical surface of IECs [17, 18]. One type is Na^+^/amino acid transport system; it transports amino acids from the intestinal lumen into cells. At least 5 amino acid transporters are present at the serosal (basolateral) surface, which can transport amino acids out of the cells and into interstitial fluid [17] (Figure 2). The amino acids can then enter blood vessels for circulation. The undigested and unabsorbed substances enter the large intestine.

### 2.2. Amino Acids Regulate the Proliferation and Differentiation of IECs

The maintenance and growth of intestine is driven by the amount of luminal nutrients. High nutrient content causes increases in the cell number, villus length, and crypt depth [13, 19, 20]. In addition, the type of nutrients appears to contribute to alterations in the morphology and functions of IECs. A recent study found that L-glutamine enhanced intestinal enterocytes growth via activation of mammalian target of rapamycin (mTOR) signaling pathway, independently of AMP-activated kinase (AMPK) [21]. In another study, dietary glutamine supplementation (1% L-glutamine, wt : wt) in piglets was found to increase the intestinal expression of genes necessary for cell growth and oxidants removal (120–124%) and reduce the expression of genes that promote oxidative stress and immune activation (34–75%) [22]. High-protein diet was found to upregulate the expression of genes related to cell proliferation and chemical barrier function in rat colon [23]. This study demonstrated that high-protein diet increased the amount of undigested peptides entering the large intestine, modified the gut microbiota composition, and increased protein fermentation by bacteria, resulting in the production of numerous amino acid derived metabolites [23].

It was also found that treatment with amino acid solution (1.0 g/L threonine, 1.2 g/L valine, 1.1 g/L serine, 0.2 g/L tyrosine, and 1.6 g/L tryptophan) increased the IECs proliferation rate in mice exposed to radiation. The treatment increased the proliferation markers (e.g., Ki-67, p-Erk, p-Akt, and PCNA) and stem cells markers (Lgr5+), decreased apoptosis markers (e.g., cleaved caspase-3), and increased protein levels of sodium–hydrogen exchanger 3 (NHE3) and sodium-dependent glucose transporters (SGLT1) in the brush border membrane [24]. Mice treated with this amino acid solution displayed decreased paracellular permeability (tightening of the mucosal barrier), increased Na^+^ absorption, and improved survival when exposed to lethal dose of total-body irradiation. The results demonstrated that the better absorption of electrolytes and nutrients could be, at least partially, attributed to an increased villus height induced by amino acid solution, and that the extracellular signal-regulated kinase (ERK) pathway in IECs was activated by the amino acid solution [24].

Oral supplementation of L-glutamine (0.5%) to weaning piglets was found to increase the villus height and crypt depth, reduce oxidative stress, increase the proliferative rates of IECs, and lower the apoptotic rate of IECs [25]. In cultured intestinal porcine epithelial cells (IPEC-1), L-arginine stimulated the proliferation rate and attenuated the lipopolysaccharide- (LPS-) induced cell death [26]. In mechanistic study, the authors found that L-arginine treatment increased relative protein levels of phosphorylated mTOR and phosphorylated ribosomal protein S6 kinase-1 and decreased relative protein levels of TLR4 and phosphorylated nuclear factor kappa-light-chain-enhancer of activated B cells (NF-*κ*B) in LPS-treated IPEC-1 cells.

### 2.3. Amino Acids Regulate the Intestinal Epithelial Barrier Functions

IECs are tightly bound together in a monolayer by intercellular junctional complexes. These cell-cell connections allow the epithelium to form a barrier which separates the extracellular fluid at the luminal side of the cell from fluid at the serosal side and also prevents microbial invasion of interstitial tissues [27]. Tight junctions are a primary determinant of intestinal epithelial barrier properties and functions. Tight junctions prevent the direct diffusion of small molecules from intestinal lumen into the interstitial spaces and then into the blood vessels [28]. Disruption of tight junction structures by specific protein mutations or by aberrant signaling can be both the cause and effect of diseases [27].

In recent years, studies have established the important roles of glutamine in regulating the functions of tight junction proteins (Figure 2). In human Caco-2 cells, a classic model for studying gut barrier function, it was found that glutamine deprivation or inhibition of glutamine synthetase significantly decreased the transepithelial resistance and reduced the expression of tight junction proteins. Glutamine addition rescued the phenotype of barrier dysfunction [29]. In another study, the authors found that glutamine deprivation upregulated the phosphatidylinositol 3-kinase (PI3-K)/Akt pathway and reduced the expression of a tight junction protein claudin-1, consequently breaking down the barrier function (with decreased transepithelial electrical resistance and increased permeability) [30]. Other junctional proteins such as ZO-1, occludin, and claudins have been reported as essential effectors of perijunctional actomyosin ring-mediated tight junction regulation [31]. In a piglet weaning-related gastrointestinal infection model, glutamine supplementation (4.4%, w/w) has been found to preserve the intestinal epithelial barrier function in the distal ileum of piglets during infection [32]. Upon exposure to* E. coli*, the expression levels of claudin-1 and occludin were reduced in control-fed animals but not in glutamine-supplemented piglets. The study showed that glutamine supplementation was beneficial in mitigating the severity of infection, probably by reducing the mucosal cytokine responses and by preserving the intestinal barrier function [32]. Oral glutamine supplementation in an intestinal ischemia-reperfusion (IR) injury rats had significant increases in jejunal and ileal bowel and mucosal weight and villus height and crypt depth compared to IR-nontreated rats [33]. Supplementation with glutamate (2%) demonstrated positive effects on the growth performance of pigs fed with mold-contaminated feed, ameliorated the imbalanced antioxidant system, and reduced the mycotoxins-caused abnormalities in intestinal structure [34]. Deficiency in dietary glutamine has been reported to impair cell signaling and result in intestinal atrophy in both piglets and infants [35].

L-Arginine supplementation at a dose of 0.5% or 1% in weaned pigs was found to protect and enhance the intestinal mucosal immune function and maintain intestinal barrier integrity after* E. coli* LPS challenge [36]. In an inflammatory bowel disease (IBD) mouse model, L-arginine supplementation suppressed the dextran sulfate sodium- (DSS-) induced intestinal mucosal injury and improved IBD clinical parameters (e.g., survival and body weight loss) [37]. Dietary L-arginine supplementation also reduced the methotrexate-induced intestinal mucosal injury and improved intestinal recovery following injury in rats [38]. In ovo feeding of L-arginine has been reported to improve the development and barrier functions of small intestine of posthatch broilers, which was attributed to the activation of the mTOR pathway [39].

### 2.4. Amino Acids Regulate the Intestinal Immunity

IECs play important roles in protecting the human body from microbial infections. The intestines contain the largest number of immune cells in the body [40] and are continually exposed to a wide range of antigens and potential immune stimuli [27]. IECs can sense and respond to microbial stimulations to reinforce their barrier function and to participate in the coordination of appropriate immune responses, ranging from tolerance to antipathogen immunity. Because IECs function as a barrier between the intestinal microbiota and the host, they are important to the maintenance of the symbiotic relationship between gut microbiota and the host by constructing mucosal barriers, secreting immunological mediators, and delivering bacterial antigens. Inflammasome expressed by IEC plays important roles in mucosal immune defense and inflammation [41]. Dysfunction of intestinal epithelium can cause diseases, and some diseases cause the abnormalities in intestinal epithelium, which could worsen the complications. The majority of immunological processes occurs in the mucosa [42]. Peyer's patches are important components of the gut-associated lymphoid tissue (GALT) in the small intestine (Figure 1). The B cells usually dominate in the germinal centers of follicles and T cells scatter between follicles. The B cells in GALT play central roles in the intestinal immune responses. Abnormalities in B cell functions can cause the development of autoimmunity [43]. B-1 cells produce most of the circulating natural antibodies. They can differentiate into immunoglobulin M- (IgM-) or IgA-secreting cells and affect tissue homeostasis and the maintenance of a symbiotic mucosal microbiota through BCR dependent and independent means [43]. The absence of IgA-producing B-1 cells in the intestines might increase the risk of food allergies [44]. Regulatory T cells (Tregs) are a subpopulation of T cells. They function to suppress dysregulated immune responses of other immune cells to maintain intestinal homeostasis [45]. Tregs have been suggested to play important roles in other physiological and pathophysiological processes, including the pathogenesis of IBD, controlling gut microbial diversity, and promoting intestinal tissue remolding/repair in response to damage [45].

Adequate nutrition is essential to the development and maintenance of the immune system. Extensive studies have shed light on the homeostatic regulation of amino acids in intestinal immunity. It was found that glutamine reduced the proinflammatory IL-6 and IL-8 production in intestinal biopsies, and enhanced anti-inflammatory IL-10 level in the gut [46]. IL-10 is mainly produced by leukocytes (e.g., T cells and B cells) and also by epithelial cells. IL-10 plays important roles in the maintenance of intestinal mucosal homeostasis and in the suppression of proinflammatory responses of innate and adaptive immune cells [47]. Oka and colleagues found that compared to the regulatory B cells (Bregs) from healthy controls, Bregs from patients with Crohn's disease had impaired IL-10 production and that Bregs-depleted mice developed more severe intestinal inflammation compared to the controls. The results suggested that a decrease or loss of Bregs function exacerbated intestinal inflammation [48]. The IL-10-producing regulatory B10 cells have been shown to inhibit the DSS-induced intestinal inflammation/injury in a colitis mouse model [49].

Amino acids have been shown to affect the development, maturation, and functions of B cells and T cells in the intestine. The deficiency of arginine has been shown to impair early B cell maturation in F/A*-2*^*+/+*^ transgenic mice, leading to drastically reduced B cell numbers in small intestine and decreased serum IgM levels [50]. Studies using amino acid transporter ASCT2-deficient mice (*Slc1a5*^−/−^ mice) demonstrated that ASCT2 plays important role in the development of the T helper 1 (Th1) and Th17 responses and in the regulation of CD4 T helper cell function [51]. It was found that, due to a reduction in glutamine import, the mTORC1 signaling in T cells was reduced and the proinflammatory CD4+ T cell responses were attenuated [51]. Cobbold and colleagues found that depletion of the essential amino acid(s) inhibited the activation and proliferation of T cells and mTOR signaling in dendritic cells [52]. Glutamine has been found to be required for T cell activation. Depletion of glutamine blocked the proliferation and cytokine secretion of T cells. And, the T cell activation induced glutamine uptake and metabolism regulated by ERK/MAPK signaling [53]. Ikeda and colleagues found that branched-chain amino acids (BCAA, e.g., isoleucine) regulated the maintenance of Tregs through Slc3a2-dependent metabolic reprogramming. Mice fed a reduced BCAA diet had reduced Foxp3^+^ Tregs numbers and Foxp3^+^ Tregs in mice on a reduced leucine diet showed defective proliferation [54]. Oral supplementation of L-arginine has been shown to improve the intestinal immune function and reduce bacterial and endotoxin translocation in experimental severe acute pancreatitis rats [55]. The treatment increased CD3+ and CD4+ T-lymphocyte numbers and CD4+/CD8+ ratio in intestinal mucosal lamina propria [55].

These findings may help understand the beneficial effect of glutamine on reducing morbidity and gut permeability in patients with multiorgan system trauma [56]. In a rat model of puncture-style brain trauma, glutamine administration reduced the gut damage and decreased intestinal NF-*κ*B activity and intestinal proinflammatory cytokines expression [57]. Oral and parenteral feeding studies have demonstrated that, in addition to the total protein intake, the availability of specific dietary amino acids (particularly glutamine, glutamate, and arginine) is also essential to optimizing the immune functions of intestine [42]. Deficiencies in amino acids such as tryptophan, arginine, glutamine, and cysteine can reduce immune cells activation [58]. These amino acids have been shown to play unique roles in maintaining the integrity, growth, and function of the intestine, in normalizing inflammatory cytokines secretion, in improving T-lymphocyte numbers, specific T cell functions, and the secretion of IgA [59]. In a 2,4,6-trinitro-benzene sulphonic (TNBS) acid-induced ulceration rat model, compared to rats fed with control-diet, the rats fed with glycine showed mild ulceration and localized inflammatory cells in the mucosa and the submucosal area, decreased colonic myelo-peroxidase activities (70%), and decreased TNBS-induced IL-1*β* and TNF-*α* mRNA [60].

Studies in mice have demonstrated that dietary amino acids stimulation regulates the homeostasis of macrophages in the small intestine. For instance, when mice were given total parenteral nutrition, which deprived the animals of enteral nutrients, there was a significant decrease in IL-10-producing macrophages (e.g., F4/80+CD11b+ macrophages) in small intestine, whereas the IL-10-producing CD4+ T cells remained unchanged [61]. The production of IL-10 in small intestine is dependent on both diet and microbiota. These data demonstrated a strong association between amino acid deprivation and the impaired replenishment of intestinal immunity function; a prolonged protein (amino acids) deficiency impairs critical immune functions [61].

L-arginine has been shown to be involved in protein synthesis and in the regulation of many essential cellular functions including immune response, hormone secretion, and wound healing [62]. L-arginine (0.5%) supplementation in pigs increased the numbers of IgA-secreting cells and CD8+ and CD4+ T cells in ileum (*P* < 0.05) [36]. In human Caco-2 cells, L-arginine was found to ameliorate intestinal inflammation through attenuating Interleukin-1*β*- (IL-1*β*-) induced NF-*κ*B activation [63]. L-arginine and its metabolite ornithine promote colonic epithelial wound repair by enhancing cell proliferation and collagen deposition through activation of mTOR signaling pathway [64]. Dietary glycine and L-glutamine supplementation has been shown to protect the colonic wall of irradiated rats [65, 66].

Although prolonged protein deficiency can impair critical immune functions, short-term protein restriction has been shown to enhance immunity to pathogens [67] and regulate inflammation [68]. The integrated stress response (ISR) is a coordinated cellular program that allows eukaryotic cells to sense and response to stress-induced signals, including nutrient deprivation (e.g., amino acid starvation) and endoplasmic reticulum stress [69, 70]. General control nonderepressible 2 (GCN2) is an ISR sensor that can sense amino acid depletion [69]. A recent study revealed that acute amino acid starvation (using 2% protein diet or leucine-deficient diet) suppressed intestinal inflammation and ameliorated disease severity in a DSS-induced mouse colitis model through a GCN2-dependent mechanism [69]. The authors demonstrated that amino acid starvation activated ISR and GCN2 suppressed intestinal inflammation and Th17 responses through a mechanism dependent on autophagy, a decrease in oxidative stress, and reductions of inflammasome activation and IL-1 *β* production [69]. In a previous study, Sundrud and colleagues found that selective amino acid deprivation inhibited Th17 cell differentiation and that a small molecule halofuginone selectively inhibited Th17 differentiation by activating the amino acid starvation response pathway. The authors also found that halofuginone suppressed the Th17-associated autoimmune inflammation in two experimental autoimmune encephalomyelitis mouse models [71]. These findings illustrate the ability of T cells to sense amino acids levels in local environment, rather than using the amino acid solely as a fuel source, reveal a mechanism coupling the amino acid sensing with intestinal inflammation via GCN2, and potentially help identify novel targets for therapeutic intervention of IBD and other inflammatory diseases.

## 3. Concluding Remarks

In addition to providing the monomer units for proteins syntheses, amino acids participate in diverse cellular functions. Extensive studies have demonstrated that (1) amino acids play important roles in maintaining IECs growth, structure, homeostasis, and functions; (2) amino acids regulate the intestinal epithelial barrier structure and functions; (3) amino acids regulate the intestinal immunity functions; and (4) amino acids supplementation has the potential to ameliorate the abnormalities/phenotypes associated with dysfunctional IECs, through restoring the properties (and functions) of IECs (Table 1). These findings help further our understanding of the important roles of amino acids in the homeostasis of IECs and in maintaining the physiology of intestine, digestive system, and human body and could potentially help identify novel targets and reagents for therapeutic interventions of diseases associated with dysfunctional IECs.

## Figures and Tables

**Table 1:** The roles of amino acids (arginine, glutamine, and glycine) in several intestinal disorders.

Amino acid	Disorder	Model	Role (mechanism)	Reference
Arginine	IBD	DSS-induced mouse colitis	L-arginine supplementation improved responses to DSS-induced injury and inflammation (e.g., colonic permeability ↓, proinflammatory cytokine and chemokine expression ↓, wound repair capacity ↑).	[37]
			Dietary arginine supplementation had beneficial effects on clinical and biochemical parameters of colitis (e.g., changes in serum amino acids profile, colonic oxidative injury ↓, Claudin-1 ↑).	[59]
	Weaning-related GI infections	*E. coli* LPS challenged- weaned pigs	L-arginine supplementation improved intestinal mucosal immune function and maintained barrier integrity in response to LPS challenge (e.g., Intraepithelial lymphocytes ↑, IgA-secreting cells ↑, CD8(+) and CD4(+) T cells ↑, mast cells ↓, Peyer's patch lymphocyte apoptosis ↓).	[36]
	Radiotherapy (cancer)	Abdominal irradiation rat model	Arginine supplementation had protective effect on maintaining the colonic wall of irradiated rats (maintaining the partial volume of the colonic epithelium, not the total volume of the wall).	[66]

Glutamine	Weaning-related GI infections	Weaned piglets of *E. coli* infection	L-Glutamine supplementation reduced the severity of infections by improving intestinal barrier function (e.g., potential difference and Isc ↑, junctional proteins expression ↑), and by reducing mucosal cytokine response (e.g., IL-1*β*, IL-6, and TGF-*β* ↓).	[32]
	Intestinal IR	IR injury rat model	Oral glutamine supplementation attenuated IR-induced mucosal injury and improved intestinal recovery (e.g., enterocytes proliferation ↑, bowel and mucosal weight, mucosal DNA, villus height, and crypt depth ↑).	[33]
	TBI-associated intestinal mucosal damage	TBI rat model	Glutamine supplementation ameliorated the TBI-caused intestinal structure damage (intestinal apoptosis ↓, NF-*κ*B activity ↓, IL-1*β*, IL-6, and TNF-*α* ↓).	[57]
	IBD	Human duodenal biopsies	Glutamine has favorable effects on cytokine responses in human intestine (IL-1*β*-induced IL-6 and IL-8 ↓, IL-10 ↑).	[46]
	IBD	DSS-induced mouse colitis	Dietary glutamine supplementation had beneficial effects on the clinical and biochemical parameters of colitis (e.g., changes in serum amino acids profile, NF-*κ*B activity ↓, PI3K-Akt signaling ↓).	[59]
	Radiotherapy (cancer)	Abdominal irradiation rat model	Glutamine supplementation had protective effect on maintaining the colonic wall of irradiated rats.	[65]

Glycine	IBD	TNBS- or DSS-induced rat colitis	Dietary glycine prevented TNBS- or DSS-induced colitis by inhibiting inflammatory cytokine and chemokine production (e.g., IL-1*β*, TNF-*α*, myeloperoxidase activities, cytokine-induced neutrophil chemoattractant, and macrophage inflammatory protein ↓).	[60]
	Radiotherapy (cancer)	Abdominal irradiation rat model	Glycine supplementation had protective effect on maintaining the colonic wall of irradiated rats (maintaining the thickness of colonic wall and mucosal epithelium).	[65, 66]

*Note*. (1) ↑: increased/upregulated; ↓: decreased/downregulated; (2) DSS: dextran sulfate sodium; GI: gastrointestinal; IBD: inflammatory bowel disease; IR: ischemia-reperfusion; TBI: traumatic brain injury; TNBS: 2,4,6-trinitro-benzene sulphonic acid.

## Abbreviations

BCAA: Branched-chain amino acids
Bregs: Regulatory B cells
ERK: Extracellular signal-regulated kinase
IBD: Inflammatory bowel disease
IECs: Intestinal epithelial cells
IR: Ischemia-reperfusion
ISR: Integrated stress response
GALT: Gut-associated lymphoid tissue
GCN2: General control nonderepressible 2
GI: Gastrointestinal
Ig: Immunoglobulin
IL-1*β*: Interleukin-1*β*
LPS: Lipopolysaccharide
mTOR: Mammalian target of rapamycin
NF-*κ*B: Nuclear factor kappa-light-chain-enhancer of activated B cells
PI3K: Phosphatidylinositol 3-kinase
Th: T helper cells
TLR: Toll-like receptor
TNBS: 2,4,6-Trinitro-benzene sulphonic acid
Tregs: Regulatory T cells.
